# Neoantigen: A New Breakthrough in Tumor Immunotherapy

**DOI:** 10.3389/fimmu.2021.672356

**Published:** 2021-04-16

**Authors:** Zheying Zhang, Manman Lu, Yu Qin, Wuji Gao, Li Tao, Wei Su, Jiateng Zhong

**Affiliations:** ^1^ Department of Pathology, Xinxiang Medical University, Xinxiang, China; ^2^ Department of Gastroenterology, Cancer Hospital Affiliated to Zhengzhou University, Zhengzhou, China; ^3^ Department of Pathology, The First Affiliated Hospital of Xinxiang Medical University, Xinxiang, China

**Keywords:** immunotherapy, neoantigen, tumor-specific antigens, vaccine, personalized cancer immunotherapy

## Abstract

Cancer immunotherapy works by stimulating and strengthening the body’s anti-tumor immune response to eliminate cancer cells. Over the past few decades, immunotherapy has shown remarkable efficacy in the treatment of cancer, particularly the success of immune checkpoint blockade targeting CTLA-4, PD-1 and PDL1, which has led to a breakthrough in tumor immunotherapy. Tumor neoantigens, a new approach to tumor immunotherapy, include antigens produced by tumor viruses integrated into the genome and antigens produced by mutant proteins, which are abundantly expressed only in tumor cells and have strong immunogenicity and tumor heterogeneity. A growing number of studies have highlighted the relationship between neoantigens and T cells’ recognition of cancer cells. Vaccines developed against neoantigens are now being used in clinical trials in various solid tumors. In this review, we summarized the latest advances in the classification of immunotherapy and the process of classification, identification and synthesis of tumor-specific neoantigens, as well as their role in current cancer immunotherapy. Finally, the application prospects and existing problems of neoantigens were discussed.

## Introduction

The human immune system helps us avoid infections and many diseases and protects us from cancer (1, 2). With the ability to recognize its own and non-self substances, the body’s immune system can produce natural immune tolerance to its own components and eliminate non-self foreign bodies to maintain the internal environment’s stability (3). Cancer occurs when normal cells change and begin to lose control. Since cancer cells are derived from normal cells and are indistinguishable from normal cells, the immune system’s ability to recognize cancer cells is minimal (4, 5). Cancer cells can avoid being attacked by the immune system when the immune system mistakenly thinks tumor cells are self-components. The surveillance of the immune system is also progressively weakened by mutations in the tumor. Tumor cells that activate the immune system are gradually screened out until they produce tumor molecules that are not recognized by the immune system. This process is also known as immunoediting of tumor. In this way, tumor cells successfully escape the damage of the immune system and have a chance to develop. What’s more, because cancer cells themselves can also release many substances that block the immune system, tumor immune response is often selectively suppressed around the tumor tissue (6, 7), which explains the ineffectiveness of immunotherapy in many patients: it is the failure to activate the immune response around the tumor tissue rather than the inability to activate the immune response systematically (6–9). In addition, inflammation can promote the development of tumors. Inflammation can release a large number of immunosuppressive cytokines locally in tumor tissue and suppress the immune system through a variety of ways. So cancer still could be caused even with a normal immune system. To overcome this problem, researchers have been looking for ways to help the immune system enhance its antitumor immune responses and improve its capacity to suppress tumor. In recent years, immunotherapy has developed rapidly and become a mature cancer treatment strategy in addition to surgery, chemotherapy and radiotherapy. Immunotherapy has shown a significant therapeutic effect in many human malignant tumors by using the immune system to eliminate cancer cells (10).

With the wide application of high-throughput omics and the development of neoantigen prediction technology, immunotherapy based on neoantigen has become a new research hotspot. Neoantigens are mainly tumor-specific antigens generated by mutations in tumor cells, which are only expressed in tumor cells (11). Neoantigens can also be produced by viral infection, alternative splicing and gene rearrangement (12–14). They are ideal targets for T cells to recognize cancer cells and can stimulate strong anti-tumor immune response. Studies in the past five years have shown that neoantigens play a key role in tumor immunotherapy. The identification, screening and identification of neoantigens accelerate the development of personalized immunotherapy for tumor patients, which will benefit more patients (15). As more scientific and clinical data reveal the remarkable effects of neoantigen-based vaccine therapies in a variety of cancer types, there is ample reason to believe that neoantigen-based therapies will be a promising area of cancer immunotherapy.

## Introduction of Cancer Immunotherapy

Immunotherapy refers to the measures taken to use immunological methods and principles to target the hyper or hypo-immune state of the organism, intervene or adjust the organism’s immune function artificially, and strengthen or attenuate the immune response so as to achieve the purpose of treating diseases (16). Immunotherapy enhances the immune system’s ability to recognize, target, and eliminate cancer cells in the body, making it a potentially universal cancer solution (17). Immunotherapy, approved as a first-line treatment strategy for multiple cancers in the United States and elsewhere (18), can be used alone or in combination with other cancer treatments (19). Compared with other cancer treatments, immunotherapy has become more precise, personalized, and has fewer side effects (20–22). In recent years, it has gradually become an important development direction of cancer treatment, which is known as the fourth leading cancer treatment technology after surgery, radiotherapy, and chemotherapy.

## Classification of Immunotherapies

**Figure 1:**
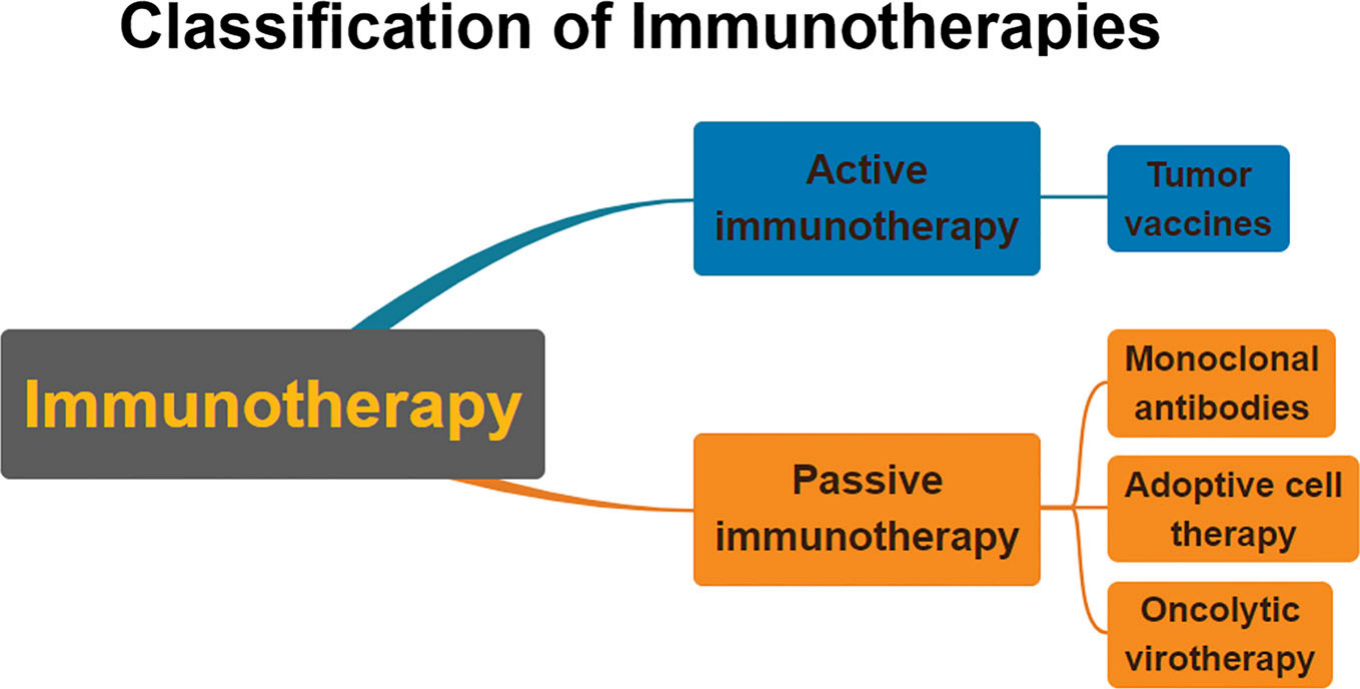
Classification of immunotherapies. Immunotherapy is usually divided into passive immunotherapy and active immunotherapy. Active immunotherapy is mainly cancer vaccines. Passive immunotherapy mainly includes adoptive cell therapy, oncolytic viruses, and monoclonal antibodies.

Immunotherapy is generally divided into two categories: active immunotherapy and passive immunotherapy (23) (**Figure 1**). Active immunotherapy refers to eliminating cancer cells by stimulating the body’s immune system (24). Passive immunotherapy refers to the passive acceptance by an organism of antibodies, cytokines, or transformed immune cells that can directly act on the tumor (25).

Tumor vaccines, one type of active immunotherapy, is an important component in the field of tumor immunotherapy (26). Tumor vaccines can recognize proteins present on specific cancer cells, arrest cancer cell growth, prevent cancer recurrence, and clear cancer cells that remain after treatment (27–30). Cancer vaccines aim to elicit immune responses against tumor-specific or tumor-associated antigens, thereby facilitating the immune system to attack cancer cells carrying these antigens (30, 31). Tumor vaccines include cell vaccines, DNA vaccines, mRNA vaccines, polypeptide vaccines, dendritic cell vaccines, and others (32, 33). In April 2010, Provenge (sipuleucel-T), the first tumor therapeutic vaccine, was approved by the FDA for the treatment of prostate cancer (34).

Monoclonal antibodies have promising therapeutic effects in the clinic and belong to passive immunotherapy (35). Monoclonal antibody drugs can specifically bind to specific receptors or ligands on the surface of tumor cells or immune cells and block the corresponding signaling pathways, thereby exerting antitumor effects (36). The hottest monoclonal antibodies in the field of oncology at present are immune checkpoint inhibitors (37, 38). Immune checkpoints are immunosuppressive pathways that they can suppress T-cell activity (39, 40), and cancer cells evade the immune response by hijacking this pathway (41–44). The most thoroughly studied immune checkpoints are CTLA-4, PD-1, and PD-L1, which have been approved by FDA for the treatment of a variety of tumors (45, 46). Adoptive cell therapy exerts its tumor-suppressive and killing effects by isolating immunocompetent cells from patients, inducing their differentiation *in vitro*, reconstituting, expanding, and re-infusing them into patients to target antigen-specific tumor cells and exert their tumor-suppressive and tumor-killing effects (47). It belongs to passive immunotherapy (48). The most promising adoptive cell therapies at present are TCR-T(TCR-modified T cell) and CAR-T(Chimeric antigen receptor T cell) (49–51). Oncolytic virotherapy is a form of passive immunotherapy. Oncolytic viruses are a class of tumor-killing type viruses that render them unable to replicate in normal tissues by attenuating or deleting viral pathogenic factors while maintaining replication and killing viability in tumor cells (52, 53). IMLYGIC, the first FDA-approved oncolytic viral drug, was genetically modified from herpes virus type 1 (HSV-1) for the treatment of metastatic melanoma (54).

There are two current successes of immunotherapy, one with PD-1 and PD-L1 mAbs derived through immune checkpoints and the other with adoptive T cell therapy (29, 55). But clinical trials have shown that although having promising potential, immune checkpoint therapies have limited efficacy in many cases, especially in solid tumors with low response rates (56). What’s more, adoptive T cells present problems such as poor persistence as well as cytotoxicity *in vivo* and can trigger an inflammatory factor storm (57–59). With the wide application of multi-omics high-throughput technologies and the development of neoantigen prediction technology, neoantigen-based immunotherapy becomes a new research hotspot. In the past five years, studies have shown that neoantigens have a promising outcome in clinical therapy. Technological advances in the identification, screening, and characterization of neoantigens will accelerate the development of individual immunotherapy for cancer patients, thereby benefiting more patients. In the future, neoantigen therapy will become an important therapeutic modality in the field of precision oncology (60).

## What Is the Neoantigen?

Gene mutations caused by genetic instability during carcinogenesis always occur in the non-coding and coding region, and the amino acid sequence changes caused by mutations in the coding region can produce proteins that are not found in normal cells. These proteins can activate the immune system and lead to the immune system’s attack on cancer cells (61). Neoantigens can also be produced by viral infection, alternative splicing and gene rearrangement. These aberrant antigens, which can be recognized by immune cells and result from mutations in cancer cell genes, are neoantigen (62). Neoantigens can be presented on the cell surface and subsequently recognized by T cells under the action of major histocompatibility complex (MHC) molecules (63, 64). Tumor antigens are divided into tumor associated antigens (TAA) and tumor specific antigen (TSA) (65). TAA is a protein expressed by unmutated genes and appears to be significantly over-expressed in tumor cells but rarely expressed in normal cells (11). Because TAAs are normal host proteins, they are subject to both central and peripheral tolerance mechanisms (35, 64). Targeting TAAs may also lead to autoimmune toxicity (39); tumor specific antigen (TSA) is a neoantigen resulting from somatic mutations and is expressed only in tumor cells but not in normal cells (66). Because normal cells do not express TSA, they are considered non-self by the immune system, neoantigen specific immune responses are not affected by tolerance. Furthermore, targeting TSAs does not easily induce autoimmunity (39). Thus, neoantigens are ideal targets for therapeutic cancer vaccines and T cell-based cancer immunotherapy. By taking advantage of the immune activity of neoantigens, synthetic neoantigen drugs can be designed according to the situation of tumor cell mutation to achieve the effect of treatment.

## Classification of Neoantigens

**Figure 2:**
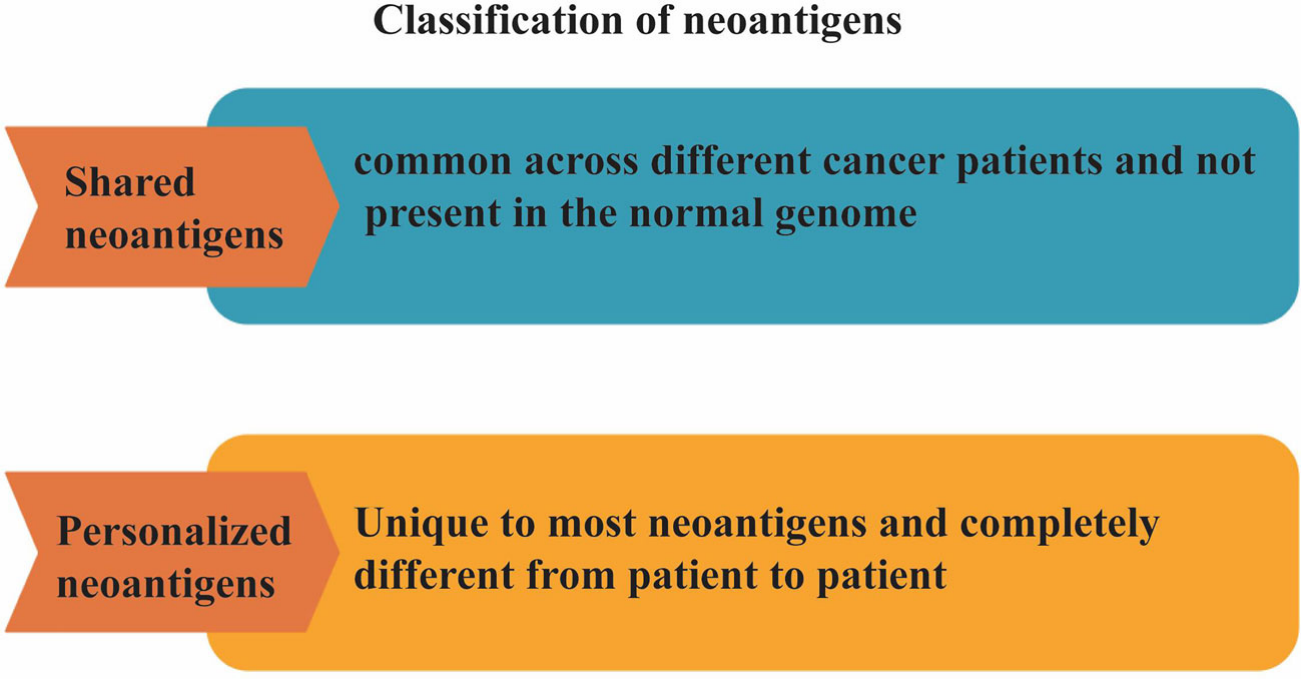
Classification of neoantigens. Neoantigens can be classified into two categories: shared neoantigens and personalized neoantigens. Shared neoantigens refer to mutated antigens that are common across different cancer patients and not present in the normal genome. Personalized neoantigens refer to mutated antigens that are unique to most neoantigens and completely different from patient to patient.

Neoantigens can be classified into two categories: shared neoantigens and personalized neoantigens (66, 67) (**Figure 2**). Shared neoantigens refer to mutated antigens that are common across different cancer patients and not present in the normal genome. Shared neoantigens that are highly immunogenic have the potential to be screened for use as broad-spectrum therapeutic cancer vaccines for patients with the same mutated gene (68, 69). Personalized neoantigens refer to mutated antigens that are unique to most neoantigens and completely different from patient to patient. Thus, the personalized neoantigen preparation drug can only be specifically targeted to each patient, that is, personalized therapy (70). Neoantigens, with strong immunogenicity, can reduces the probability of immune escape of tumor cells. However, the different types and quantities of neoantigens in different individuals of the same tumor caused by specificity of mutations showing obvious individual heterogeneity. Therefore, the application of neoantigens in tumor immunotherapy will tend to be personalized (71). Individualized cancer vaccines can work alone or in combination with other therapies to increase the strength and durability of the anti-tumor effect, improve survival and quality of life, and ultimately improve the outcome of cancer treatment for patients (72). The feasibility, safety and immunogenicity of individualized cancer vaccine in the treatment of cancer patients determine that it will be an important development trend in the future (15). It is expected that individualized cancer vaccines will enable most patients to obtain precise treatment in the foreseeable future.

## Identification of Neoantigens

**Figure 3:**
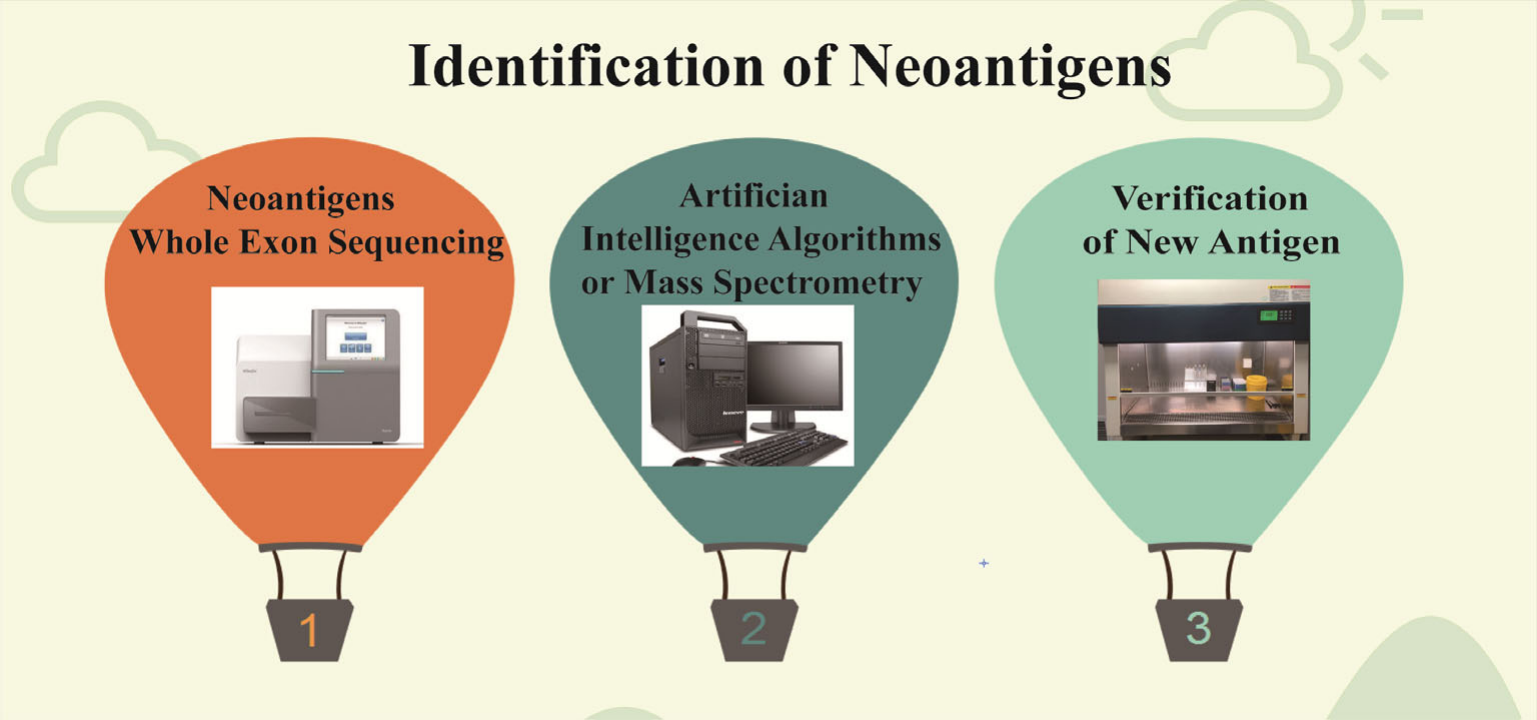
Identification of neoantigens. First, the new antigens were found by high-throughput sequencing, then screened by algorithm and mass spectrometry, and finally verified by experiment.

Although neoantigens have made good clinical progress in tumor therapy, the number of neoantigens with immunogenicity is small, and the prediction comparison is difficult (73). Therefore, the neoantigen field needs more optimized algorithms and validated methods for accurate prediction in order to select more reliable neoepitopes of high immunogenicity. At present, the prediction accuracy of tumor neoantigen remains an urgent problem. For tumor neoantigen prediction algorithm, there are many factors to be considered, including HLA typing, expression, mutation analysis, prediction peptide processing, TCR binding force, MHC affinity, PMHC stability, tumor neoantigen source,and so on (74, 75). It also includes T cell recognition, TCR analysis, and immune cell analysis to assess T cell response (74, 76). For neoantigen screening and assessment of T cell responses, in addition to next-generation sequencing, there are high-resolution and tandem mass spectrometry techniques as well as in silico techniques for peptide prediction, but prediction algorithms based on machine learning and AI techniques need to be continuously trained with confirmatory datasets where data type, quality, and quantity can greatly affect algorithm precision (77, 78) (**Figure 3**). Therefore, the continuous accumulation of databases, especially the validated tumor neoantigen data, is extremely critical to improving algorithm accuracy (79, 80). The tumor neoantigen selection Alliance (TESLA) was initiated and formed by the Park Institute for cancer immunotherapy (PICI) and the Cancer Research Institute (CRI) (81). TESLA brings together 36 top biotechnology, pharmaceutical, university, and non-profit research teams, consisting of the National Cancer Center (NCC), PICI, Memorial Sloan-Kettering Cancer Center (MSKCC), MD Anderson Cancer Center, and more than 30 other top neoantigen research institutions. The consortium aims to establish algorithms and standards for global neoantigen testing, make concerted efforts to predict more precise anticancer targets, and advance research and application of personalized tumor vaccines. TESLA scientists discovered algorithmic models and core parameters that can better predict neoantigens and accurately predicted 75% of validated neoantigen targets and filtered to exclude 98% of invalid neoantigen targets, whose findings were published in cell journals (81).

## Neoantigen Synthesis Process

**Figure 4:**
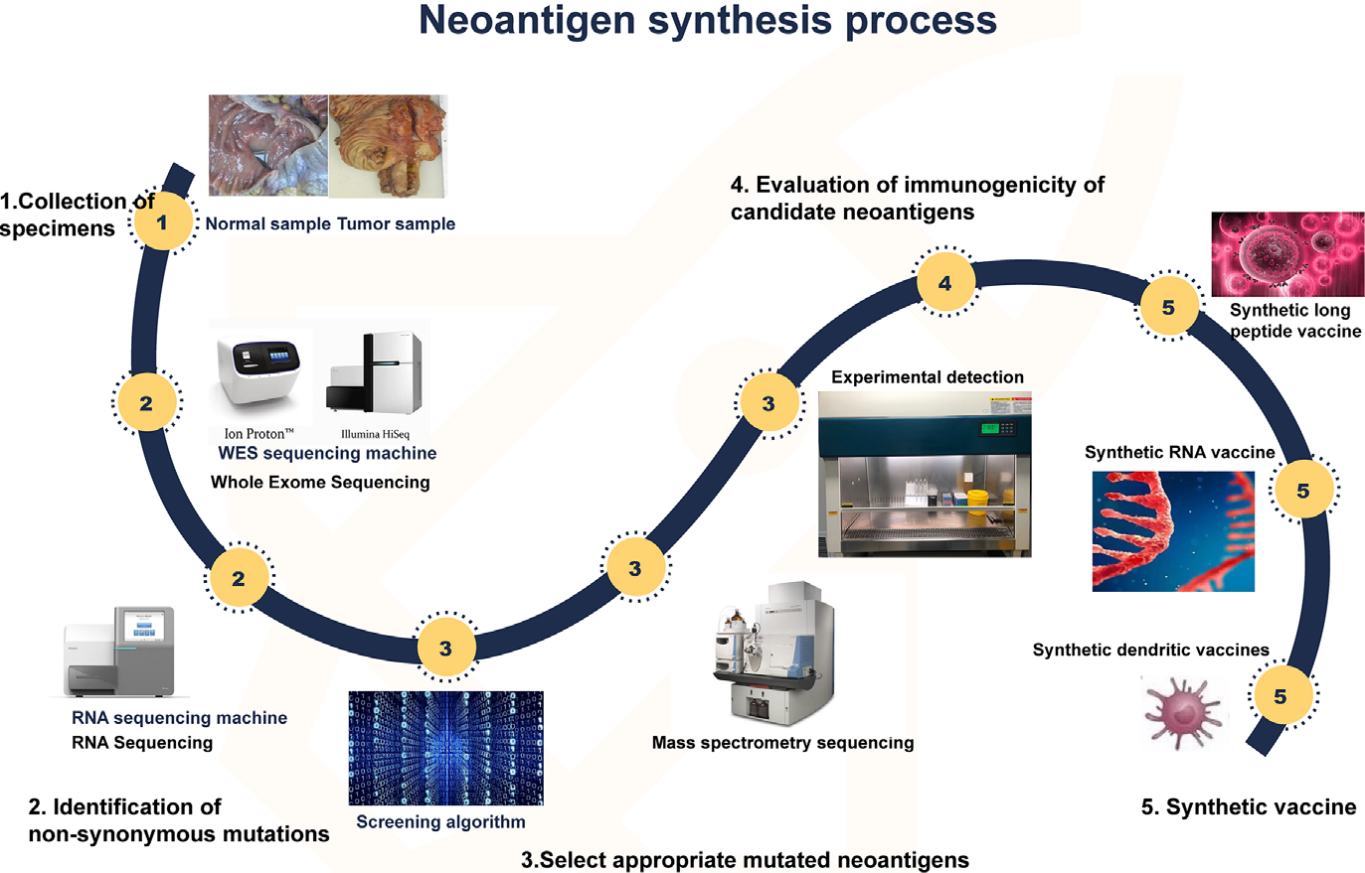
Synthesis of neoantigen. First, tumor and normal tissue samples were obtained. Then, by comparing the sequencing results of the two groups of samples, the mutated gene of the tumor was identified. Using computer, mass spectrometry or experimental methods to screen the gene sequences that are most likely to become tumor neoantigens, and finally these mutated genes can be designed into vaccines, which can take many forms, such as peptide vaccines, dendritic cell vaccines, RNA vaccines, etc.

The development strategy for tumor neoantigen vaccines is clear. First, obtaining tumor tissue and normal tissue samples from the patient and then identifying the mutant genes of the tumor by comparing the exome sequencing results of the two groups of samples. cDNA microarray or RNA sequence test was performed to select appropriate mutated neoantigens according to gene expression level. Computer analysis is used to predict the adhesion affinity of candidate antigens to HLA, and the gene sequences that are most likely to become neoantigens of tumors are screened. Finally, these mutated genes are designed into vaccines (82, 83). Cancer vaccines can come in many forms, such as peptide vaccines, dendritic cell vaccines, mRNA vaccines, DNA vaccines, and viral vaccines (84) (**Figure 4**). Different forms of vaccines have different advantages and disadvantages (**Table 1**).

**Table 1:** Advantages and disadvantages of different forms of vaccines.

Vaccines type	Advantages	Disadvantages
mRNA vaccines	1. Cellless production; 2. Delivered to DC efficiently	Easy degradation
DNA vaccines	1. Cellless production; 2. The cost is low; 3. Encode any epitope	There is a risk of mutation
Peptide vaccines	1. Cellless production; 2. Easy to transport; 3. Fully degradable	Degradation produces an unrelated immune response
Dendritic cell vaccines	1. The immune stimulation activity is strong; 2. The clinical effect has been proved	Cost is high

## Advances in the Study of Neoantigens

In 2014, Rosenberg reported in Science that he had successfully treated a patient with advanced cholangiocarcinoma, a highly aggressive form of the disease, using lymphocytes that were amplified *in vitro* and specifically recognized cancer cells (85). Whole exome sequencing revealed a total of 26 significant gene mutations in the patient, many of which produced abnormal proteins. By co-culturing these abnormal protein fragments with lymphocytes isolated from the patient’s tumor tissue, CD4 positive T cells were found to recognize one abnormal protein, ERBB2IP. Lymphocytes that recognize the aberrant protein produced by the ERBB2IP mutation were expanded, activated, and returned to the patient. After two regurgitation, the lesion shrank significantly throughout the patient’s body, leading to complete remission and complete disappearance of the tumor. Cholangiocarcinoma is an extremely diffificult disease, and this patient achieved complete remission with lymphocyte infusion for several years after the failure of conventional treatments, such as chemotherapy. This report foreshadows the future role of neoantigens in immunotherapy. In 2015, Rosenberg team also reported the discovery of multiple neoantigens in the tissues of patients with digestive tract tumors, including the famous neoantigen derived from KRAS (G12D) mutation (86).

At present, a large number of neoantigens have been discovered, which are unique to cancer cells and recognized by T cells as heterogeneous, and are not affected by immune tolerance mechanism. Therefore, these antigens can be used as effective targets for immune-mediated tumor therapy. In 2017, Ugur Sahin’s team reported in Nature that a personalized RNA-based vaccine treatment regimen was used in a trial involving 13 melanoma patients. The therapy predicted the antigen of the mutations detected on each patient’s genome and then synthesized a personalized vaccine. Eight of these patients have had no further tumor recurrence over 23 months, five of them with advanced malignant melanoma, two of whom experienced significant tumor shrinkage, and one additional patient treated with the vaccine combined with a PD1 antibody had a complete tumor response. This study demonstrated the clinical feasibility, safety, and antitumor activity of personalized RNA vaccine based on multi-omics (87). In the same year, the vaccine produced by Catherine J. Wu’s team using Neoantigen also successfully treated malignant melanoma. Four of the six patients who received the vaccine were free of relapse 25 months after the vaccine, while two patients with recurrent disease were subsequently treated with anti-PD-1 therapy and experienced complete tumor regression (88). Personalized vaccines may be an important way to conquer cancer.

Genocea Biosciences’s GEN-009 vaccine trial (NCT03633110) is one of the few Phase 1/2a clinical trials that have shown the best efficacy among the current neoantigen personalization therapies with an estimated completion date of December 2022. The purpose of this study was to evaluate the safety, tolerability, immunogenicity, and antitumor activity of the personalized vaccine GEN-009 for the treatment of patients with solid tumors, which is targeted at a broad range of cancers. The results so far show that 40 doses of the vaccine have been administered, and only a few patients have experienced mild local discomfort caused by the vaccine adjuvant with no dose limited toxicity (DLT) occurred. Ninety-nine percent of the peptides selected for the vaccine produce an immune response, and so far, no patients who have received the vaccine have relapsed (73, 89).

In 2019, Patrick Ott reported in the journal Cell the results of a personalized neoantigen vaccine NEO-PV-01 combined with PD-1 blockade in patients with advanced melanoma, non-small cell lung cancer, or bladder cancer. No treatment-related serious adverse events were observed in 82 patients, and T cell responses were observed in all patients, with no obvious toxic and adverse reactions (90).

A novel bispecific-specific antibody therapy targeting common mutations in TP53 and Ras was reported in Science and Science Immunology in 2021 by Bbert Vogelstein and his team. We all know that TP53 and RAS are important tumor-related genes *in vivo*, which are often mutated. However, p53 and Ras are mainly intracellular, so antibody-based therapy cannot be achieved with conventional methods. However, proteins are degraded by the proteasome into peptides, some of which form complexes with human leukocyte antigen (HLA) proteins and are presented on the cell surface. They have developed a specific bispecific single-chain diabody (SCDB) antibody that targets TP53 and RAS mutations. This bispecific antibody can specifically recognize and activate T cells in vitro and in mice, exerting good anti-tumour effects without cross-reactivity and with a good safety profile (91, 92).

We searched ClinicalTrials.gov (https://clinicaltrials.gov/) and found 77 cases of neoantigen studies, many of them have shown the good application value of neoantigen (Search term is “neoantigen”). However, the study of neoantigen therapy starts relatively late and still in the laboratory stage, and there are no related products on the market at both home and abroad.

## Prospects and Challenges

As the incidence and mortality of cancer continue to increase, people’s desire to conquer cancer is more and more urgent. Neoantigen vaccine has shown an obvious effect on tumor treatment in clinical trials and is expected to become an important drug for alleviating the increasing tumor morbidity and mortality in the future. It has attracted the attention of experts in immunotherapy and is a significant development direction in the future. However, there are still some restricting factors in the development of neoantigens, and solving these problems is the key to the widespread popularization of neoantigens.

1. Scarce amount of antigens. Thousands of mutations in non-synonymous genes are typically found in tumor samples, but only a few ultimately meet the antigen criteria. Finding more effective antigens is a problem that needs to be solved. Studies have shown that most specific antigens tend to be distributed in non-coding regions. The development of non-coding research in recent years will also provide assistance for the discovery of new antigens.
2. Screening methods for predicting neoantigens need to be improved. The lack of effective screening methods for neoantigen is another obstacle to the development of neoantigen therapy. At present, the algorithm of predicting neoantigen is in full bloom. With the development of bioinformatics technology, artificial intelligence and machine learning, we believe that this problem will be solved soon.
3. The development cycle of neoantigen vaccine is too long. The long development cycle of neoantigen vaccine is recognized as a primary obstacle to the application of vaccine. The long development cycle leads to the increase of research and development costs, and the great pressure on laboratories and enterprises is not conducive to the clinical application of vaccines. Patients participating in the trial or treatment have a short survival period and a long development cycle, which may lead to some patients being unable to accept the final treatment due to the long drug development cycle.
4. Preparation and delivery of vaccines remains a challenge. Currently, many methods have been developed for the preparation, formulation and delivery of different cancer vaccines. Technically, however, these vaccines need to be manufactured under GMP conditions. The biggest challenge, especially for small nucleic acid therapies such as mRNA/DNA, is delivery technology.
5. The heterogeneity of the tumor is difficult to resolve. Tumors are heterogeneous in the course of evolution, so each part of the gene that may mutate is different. So it may be a paradox to get local tumor tissue from a patient in the first place to predict the neoantigen of that patient. It is still a difficult problem to use 1-2 specific neoantigens to fully recognize and kill solid tumor tissues.
6. Expensive. Therapies based on neoantigens are mostly personalized, and from initial gene sequencing to validation and production, the cost of treatment can be very high. Cost remains the biggest challenge.

## Author Contributions

ZZ has written the review. JZ and WS supervised the program. ML, YQ, WG and TL have discussed and edited the manuscript. All authors contributed to the article and approved the submitted version.

## Funding

This work was supported by the National Natural Science Foundation of China (No. U1804173, 81802470 and 81702891), Zhongyuan Qianren Jihua of Henan Province (No. ZYQR201810153), Joint construction project of Henan Medical Science and technology research plan (No. LHGJ20190452), Natural Science Foundation of Henan Province (No. 202300410326), Xinxiang Medical College research funding (No. XYBSKYZZ201632).

## Conflict of Interest

The authors declare that the research was conducted in the absence of any commercial or financial relationships that could be construed as a potential conflict of interest.
